# On Atmospheric Corpuscles

**Published:** 1861

**Authors:** F. M. Pouchet


### ON ATMOSPHERIC CORPUSCLES.

By F. M. POUCHET.

I have thought for a long time that the study of the bodies conveyed by the air into the respiratory passages of animals would offer interesting physiological results, and throw considerable light upon the subject of atmospheric micrography. Nor have I been deceived in this. In fact, in almost every class of animals, the examination of the respiratory apparatus clearly reveals the various modifications of the medium inhabited by them. But it seemed to me that the most important notions on this subject would be presented in those animals in which the air penetrates most deeply into the organism. Birds, consequently, have become the objects of particular attention, seeing that in them air, after traversing the lungs, pervades not only the different cavities of the trunk, but reaches also the interior of the esseous system. In these animals I have devoted particular attention to the examination of the bones which contain most air, and chiefly to the *humerus*. And as in these situations the corpuscles, once introduced, escape only with great difficulty, owing to the immobility of the walls and the irregularities of their anfractuosities, we there find ample vestiges of all the matters conveyed by the air into the respiratory organs.

The examination of animals living in midst of towns, and in the interior of our dwellings, will excite surprise by the enormous quantity of starch-grains contained in their respiratory organs. In birds, corpuscles of this nature will be discovered in great abundance, even in the interior of the bones, and together with them will be observed, in profusion, particles of sooty matter, and filaments derived from the various fabrics of which our clothes are made. But the further the creature lives from towns, the more remote and wild its habitation, the more rare also become all these corpuscles in the inspired air. Under these circumstances, scarcely any traces of the sort can be observed. Frequently even not a single particle of the kind in question will be observed in animals or birds living altogether in the midst of forests; in these animals, on the other hand, the whole respiratory apparatus is filled with abundant *debris* of plants,—epidermis, chlorophyll, etc.

The amylaceous particles disseminated either in the atmosphere or in the interior of animals present two conditions—they are either of the normal state or cooked. In the majority of cases, the starch is found in the former condition; but, nevertheless, we meet in the atmosphere, and in all the cavities of animals, into which the air enters, with starch-grains either simply swelled or entirely burst, by the action of heat. The latter certainly proceed only from minute particles of bread carried about by the movements of the atmosphere.—The panified starch is readily recognized by its enormous size and ruptured condition, and by the action of Iodine, which does not produce in it the same bright color às it does in ordinary starch-grains.

The birds which inhabit the interior or live in the close vicinity of towns do not obtain this abundance of amylaceous particles simply from the air they inspire; they derive, besides this source, an abundant supply from the foliage of the trees amidst which they pass part of their lives. In fact, on examining the surface of the leaves of trees in the neighborhood of cities, when they have not been washed for some days by rain, abundance of specimens of every sort of corpuscles carried in the atmosphere will be found on them, and, universally, a considerable quantity of starch-grains, together with sooty and silicious particles. On a single leaf of a horse-chestnut growing in the garden of Ecole de Medecine at Rouen, I have counted about thirty grains of wheat-starch either in the natural or panified condition.

The search for atmospheric corpuscles, in the respiratory passages is easily made. It consists simply in the passing of a stream of water through these passages, and the collection and examination of the fluid. For this purpose I inject the trachea, by means of a syringe, and when the lungs are distended with water, make incisions into them, and carefully collect all the fluid that escapes, repeating the injection several times.

In birds, I inject the trachea, and when the water has traversed the lungs and filled all the air cavities of the body, I open the thoracic cavity, and collect the liquid which escapes in a jet. In all the experiments the fluid is received in conical vessels with a narrow bottom, and when sufficient time has elapsed to allow all the corpuscles to subside, these are removed by means of a very slender pipette, and submitted to microscopic examination. The atmospheric corpuscles may be collected from the hollow bones by the same mode of procedure. To effect this, I insert the tube of a syringe into the orifice by which the air penetrates into the cavity, and then make a section of the bone at the opposite end. The water injected, at first gently and afterwards with great force, in order to carry along with it the smallest corpuscles, is received in champagne-glasses and examined. Studied in this way, the respiratory organs afford a faithful idea of the life of the animals. Not only does the examination reveal to us what sites of habitation the animals prefer, and their kind of food, but even, when they are domesticated, the profession followed by their owners.

I have found in the air-passages of man the same atmospheric corpuscles as are met with in animals. In the bodies of two persons, who died in one of our hospitals, a man and a woman, whose lungs I injected, I found a large quantity of wheat-starch, either normal or panified; particles of silex and of glass; fragments of dye wood of a beautiful red color;—fragments of dress, lastly a larva of a microscopic arachnidean, still living.

It was rational to conclude that, at certain times, the expectoration should contain corpuscles, similar to those I have described in the lungs. And this is actually the case; I have here met with normal and panified starch-grains, particles of soot, the debris of plants, filaments of wool or cotton of various colors, particles of silex, etc.

A fowl, brought up in a paved court at Rouen, afforded in its respiratory sacculi an enormous quantity of wheat-starch, normal and panified. Besides which they contained numerous filaments of cotton and linen, and an abundance of sooty particles: there were but very few siliceous grains, a circumstance probably owing to the habitation in which the bird had existed. The humerus of this bird also contained much starch, particles of soot, a considerable number of cotton and linen filaments, and even some grains of potato-starch and of glass.

Thinking that in animals, in localities where starchy matters formed an object of trade, the abundance of amylaceous particles would be still greater, I procured two young chickens which had been kept for two months by a baker. My surmise was not unfounded. The whole of the respiratory organs in these chickens, notwithstanding their youth, contained an amount of starch surpassing that which I had found in the fowl.

A pigeon taken from a dovecote in the middle of the town presented, in its respiratory passages, besides particles of silex and soot, the debris of stuffs of various colors, and grains of potato-starch, together with considerable amount of wheat-starch of all sizes, and above all, an enormous quantity of lentil-starch. Even the *humeri* contained so much of the latter that from eight to ten grains were found in every case. I was unable to explain the presence of such an abundance of lentil-starch in a bird which always swallows seed without bruising it. But I very soon discovered the source on examining the floor of the dovecote. This was completely covered with the dung of the pigeons, containing an enormous quantity of this sort of starch, which had passed through the intestines unaltered. In flying about in their dwelling the birds diffused this in the air, and it thus gained an entrance into their respiratory organs.

The examination of a bird which is ordinarily kept only in wealthy establishments, affords another proof of what has been said. In fact, the numerous vestiges of magnificent stuffs exhibited in its respiratory organs manifestly recalled the luxurious dresses or works of those amongst whom it had lived. This bird was a peacock. Unfortunately I had at my disposal only its humeri; but having injected them, I was really struck with the abundance of, and the splendid colors presented by, all the fragments of stuffs contained in these bones. I found besides a considerable quantity of wheat-starch, numerous filaments of wool and of silk of the most magnificent blue, of a beautiful rose, and bright green.

The lungs of a mouse also afforded starch, silex and soot, but in far less quantity and in far smaller fragments, than in birds.

But if our attention is directed to wild birds, residing at a distance from cities, we observe a totally different thing.

A grey falcon (*Falco cinereus*, Mont.) killed in a large forest two leagues from any habitation, did not afford the least trace of starch, either in its air passages or within the bones. There were met with only a few particles of soot and silex;—and not a single filament of any kind of tissue was recognized. But, on the contrary, all the air-passages were filled with an abundance of the detritus of plants and debris of insects.

In another wild bird (Picus viridis, Linn.) I found in the air-passages only an insignificant quantity of starch, and very little soot and silex.

In some frogs taken in the basins of the Jardin des Plantes, at Rouen, which is situated close to numerous factories, and in a populous quarter, the lungs have always afforded a notable quantity of starch, an abundance of particles of charcoal and coal-soot, together with numerous fragments of silex and vegetable debris. Besides these, filaments of cotton, raw or manufactured, were extremely abundant. The respiratory organs of these animals also contained Naviculæ, diatoms, papilionaceous scales, the stems of mucedinous fungi, and fragments of confervæ.

If, again, we explore the respiratory passages of some animals, which, although living in a state of liberty, are in the habit of frequenting our dwellings, we find in them evident vestiges of their double existence, wild and domestic.

A jackdaw afforded a striking instance of this. Its respiratory organs contained a very considerable quantity of wheat-starch; what was very remarkable, an enormous number of sooty particles—a circumstance which is accounted for by the almost habitual abode of this bird on the lofty buildings of towns. There were found also in its air-sacs, numerous filaments of cotton and abundant debris of plants.

In all my observations, which, without exaggeration, might be counted by hundreds, I have never met with either a single spore or a single ovum of a microzoon, nor with any encysted animalcule. Moreover, in all these minute researches I have always been able to detect starch-grains wherever they existed. Is it possible that the atmospheric spores and ova alone should have escaped detection? The ova of certain Paramœcia, being .0420 mm. in diameter, and consequently surpassing considerably in bulk the largest grains of wheat-starch, whose diameter does not exceed .0336 mm., if they really existed in the atmosphere in sufficient quantity to explain the generation of Infusoria, whose apparition astonishes and stupefies us, should have been immediately discovered in the same situations, and far more easily even than the starch-grains, seeing that they ought to exist in much greater numbers. To a negation of this kind, in the actual state of science, but one answer is possible—*show these ova.*—*Comptes Rendus*, 1, 1860.—*Quarterly Journal of Microscopical Science.*

